# Single-Base Resolution Map of Evolutionary Constraints and Annotation of Conserved Elements across Major Grass Genomes

**DOI:** 10.1093/gbe/evy006

**Published:** 2018-01-25

**Authors:** Pingping Liang, Hafiz Sohaib Ahmed Saqib, Xingtan Zhang, Liangsheng Zhang, Haibao Tang

**Affiliations:** 1 Key Laboratory of Genetics, Breeding and Multiple Utilization of Corps, Center for Genomics and Biotechnology, Ministry of Education; Fujian Provincial Key Laboratory of Haixia Applied Plant Systems Biology, Fujian Agriculture and Forestry University, Fuzhou, China; 2 Key Laboratory of the Ministry of Education for Coastal and Wetland Ecosystems, College of the Environment and Ecology, Xiamen University, China; 3 Institute of Applied Ecology, Fujian Agriculture and Forestry University, Fuzhou, China; 4 State Key Laboratory of Ecological Pest Control for Fujian and Taiwan Crops, Fujian Agriculture and Forestry University, Fuzhou, China

**Keywords:** conserved noncoding sequences, synteny, purifying selection, phylogenetic footprinting, comparative genomics

## Abstract

Conserved noncoding sequences (CNSs) are evolutionarily conserved DNA sequences that do not encode proteins but may have potential regulatory roles in gene expression. CNS in crop genomes could be linked to many important agronomic traits and ecological adaptations. Compared with the relatively mature exon annotation protocols, efficient methods are lacking to predict the location of noncoding sequences in the plant genomes. We implemented a computational pipeline that is tailored to the comparisons of plant genomes, yielding a large number of conserved sequences using rice genome as the reference. In this study, we used 17 published grass genomes, along with five monocot genomes as well as the basal angiosperm genome of *Amborella trichopoda*. Genome alignments among these genomes suggest that at least 12.05% of the rice genome appears to be evolving under constraints in the Poaceae lineage, with close to half of the evolutionarily constrained sequences located outside protein-coding regions. We found evidence for purifying selection acting on the conserved sequences by analyzing segregating SNPs within the rice population. Furthermore, we found that known functional motifs were significantly enriched within CNS, with many motifs associated with the preferred binding of ubiquitous transcription factors. The conserved elements that we have curated are accessible through our public database and the JBrowse server. In-depth functional annotations and evolutionary dynamics of the identified conserved sequences provide a solid foundation for studying gene regulation, genome evolution, as well as to inform gene isolation for cereal biologists.

## Introduction

Comparative genomics is an important tool to identify both coding and regulatory DNA elements in the genome—based on the premise that these elements are under purifying (negative) selection that resist mutations during evolution (Tagle et al. 1988; Duret and Bucher 1997; Freeling and Subramaniam 2009). Nonfunctional or neutrally evolving sequences are expected to diverge faster than the sequences under selective constraints (Carginale et al. 2004; Clauss and Mitchell-Olds 2004; Zhang et al. 2004; Reineke et al. 2011). Patterns of sequence conservation among closely related species may be used to identify “footprints” of the noncoding regulatory elements to infer their functions (Nelson and Wardle 2013). The related methods are collectively called “phylogenetic footprinting” (Boffelli et al. 2003).

Many conserved noncoding sequences (CNSs) are known to function as *cis*-regulatory elements which are involved in the regulation of transcription and modulation of chromatin structure (Venkataram and Fay 2010; Raatz et al. 2011; Zhang et al. 2012; Zhang, Yuan, et al. 2016). CNSs are found to function as regulatory regions—such as the transcription factor binding sites and enhancers (Hardison 2000; Turco et al. 2012). For example, bilateral conserved regulatory elements (*Bicores*) (Clarke et al. 2012) are involved as enhancers in the vertebrate central nervous system, and are known to drive expression of transcriptional factors like *Atf*. Those CNSs act like switches of expressed genes, which can turn “on” and “off” of the expression of particular genes at specific development stages or in specific tissues. Moreover, they may be involved in the regulation of posttranscriptional process as sequences of microRNAs or small nucleolar RNAs. For instance, microRNAs can suppress translation of target genes by binding to their mRNA and the bipartite coupling of microRNA-target can be preserved over evolutionary time (Bentwich et al. 2005; Friedman et al. 2009). Accelerated evolution of CNSs, which occurs disproportionately near the genes with particular biological functions, may contribute to the divergence of species (Prabhakar et al. 2006). Deletions of CNSs could also lead to the divergence of species, with one example of such deletions involves a forebrain subventricular zone enhancer near the tumor suppressor gene that detains growth and DNA-damage-inducible gamma (GADD45G) (Zerbini et al. 2004; McLean et al. 2011). The deletion of this noncoding enhancer sequence is closely associated with the expansion of specific brain regions in humans (McLean et al. 2011).

In contrast to the many examples in animal studies, specific functions of most of the CNSs found in plants are still largely unknown to date due to relatively slow progress in annotation of such sequences (Freeling and Subramaniam 2009). In both plants and mammals, regulatory genes tend to have higher association with CNSs, such as genes that are enriched with various transcription factor binding motifs, than other classes of genes (Kaplinsky et al. 2002; Buchanan et al. 2004; King et al. 2005; Siepel et al. 2005). Regulatory genes in plant genomes tend to be associated with fewer and shorter CNSs when compared with the mammalian genes at similar divergence level and also tend to degrade faster than mammalian CNSs over evolutionary time (Inada et al. 2003; Reineke et al. 2011).

Despite the differences in lengths, CNSs present in plant and animal genomes still share functional characteristics (Strähle and Rastegar 2008; Burgess and Freeling 2014). Since the overall structure of most mammalian genomes are more conserved, comparative genomics on mammal have made substantial progress. It has been estimated that ∼3.5% of the human genome is presumed to be comprised of noncoding sequences involved in various gene regulatory processes (Drake et al. 2006). There are many examples of genes enriched with CNSs that are more likely to be retained under evolutionary selection. Genes enriched with CNSs are more likely to be subfunctionalized following gene duplication (Force et al. 1999) or selection against disruption of DNA-protein stoichiometry (Schnable and Freeling 2011).

In recent years, plummeting cost of sequencing has led to a wealth of sequenced genomes, which has accelerated the development of sophisticated methods and software to identify the CNSs by genome-wide comparison of closely related genomes and to predict the regulatory functions of those CNSs. Sequence conservation is a useful metric to identify functional coding as well as noncoding regions of the genome (Loots et al. 2000; Woolfe et al. 2005; Wang et al. 2009). Although there are many studies related to conserved sequences of protein-coding regions which include various conserved protein domains (Haudry et al. 2013; Hupalo and Kern 2013), study of noncoding sequences is still daunting task and mostly understudied, especially in plants.

The grass family (Poaceae) are the fifth largest plant family with ∼12,000 species of monocotyledonous flowering plants (Soreng et al. 2015). It is also one of the most economically important plant families, including many grain crops such as rice, wheat, barley, maize, millet for human and forage consumption, and building materials such as bamboo. Grass genomes share lots of similarities in synteny and collinearity, and could be considered a “single genetic system” (Freeling 2001). Studies have reported that critical mutational sites that are of agronomic significance of many cereal crops occurred in the conserved regions during domestication (Tang et al. 2010). One such example is the gene that controls the seed shattering trait in several grain crops (Houston et al. 2013; Tang et al. 2013; Wang et al. 2015). Identification of CNSs through comparative genomics is a valued resource, which will provide additional functionally sequence sites that have the potential to become future targets for crop engineering.

Recently, it has become possible to perform the comparative genomics in Poaceae on a large scale as the number of sequenced genomes in Poaceae have greatly increased. Whole genome comparisons of another important crop clade, including many of the crucifer genomes, identified and characterized over 90,000 CNSs with a large proportion of CNSs predicted to be involved in transcriptional and posttranscriptional regulation (Haudry et al. 2013).

In prior studies, several methods were developed to identify functional elements through the scoring of sequence conservation, such as GERP (Cooper et al. 2005) which is primarily a column-by-column method, where column represents an aligned base in the multiple sequence alignments. Such scoring method was shown to be effective for mammalian genome sequences and conceptually similar to PhyloP (Pollard et al. 2010). Another method, PhastCons (Hubisz et al. 2011), models the genome as one of the two states, one state of conserved region and one state of nonconserved region. Each state has different substitution rate parameters where PhastCons seeks to estimate while simultaneously considering phylogenetic relationships and sequence similarity using a Hidden Markov Model (HMM).

Herein, we report the genome-wide high-resolution atlas of noncoding regions under selection in the publicly available grass genomes and other related nongrass monocot genomes serving as “outgroups.” To our knowledge, this is the largest genome-scale CNS mining efforts conducted across a set of plant genomes to date. We identified and characterized CNSs in different major clades, providing several “tiers” of conservation at various divergence levels including the family, order, or the clade level. Finally, we have released the curated CNS elements through our public database and interactive genome browser tools.

## Materials and Methods

### Whole-Genome Alignments

In this study, we used 17 grass genomes, 5 monocotyledons of nongrass monocot genomes, and *Amborella trichopoda*, all of which has relatively high quality full genomes sequences available. The genome sequences information is listed in supplementary table S1, Supplementary Material online. Coding sequences (CDS) align refers to the overlap of alignment with existing rice (*O. sativa ssp. japonica*) CDS annotation as determined by intersections using BEDTOOLS (Quinlan and Hall 2016). Due to polyploidization in plants that created a large number of regions that are paralogous, we found an optimal set of local alignments that aim at retrieving orthologous alignments that are descended from the same sequence in last common ancestor of the genomes, through rigorous filtering of all sequence alignments.

Each genome was split by chromosome or contig sequences for parallel computation and aligned to *O. sativa ssp. japonica* (reference genome) using LAST v759 (Frith and Kawaguchi 2015). The LAST parameters that we used were: *lastdb* option “-uMAM8,” and *lastal* options “-p HOXD70-e4000-C2-m100.” Simple repeats were identified using *tantan* (Frith 2011) which was built in LAST in order to finding orthologous sequences more accurately. Alignments generated by LAST were linked into longer chains using axtChain (Kent et al. 2003), with chains scored <1,000 removed to retain only the significant alignments. The long chains were then assembled into longer stretches of synteny by chainNet (Kent et al. 2003). We then extracted the sequences based on the coordinates indicated in the chain and net files. After all the pairwise alignments between each of the 22 nonreference genomes against the rice reference, we used ROAST (reference dependent multiple alignment tool) to join the pairwise alignments of each nonreference genome to the rice reference genome according to the tree topology in figure 2. Our CNSpipeline is consisted of a set of Python scripts for performing individual steps and is available on GitHub: https://github.com/liangpingping/CNSpipeline, last accessed January 13, 2018.

### Calculation of Conservation Score for Every Base and Identification of CNSs in Each Clade

We estimated a simple “conservation score” of every single base. The score is based on the number of species matched to this site of reference genome, divided by the total number of species that are being compared. For example, a score of 1 suggests that the site is ultraconserved across all species, while 0 suggests that this site is not seen in other species. Additionally, we aggregated the average conservative score across each gene. CNSs were identified as fragments located beyond the coding sequences in the reference genome that showed score of at least 0.7. Lastly, merged fragments having distance within 3 bp, and we then removed the fragments that are shorter than 6 bp.

These parameters were selected based on comparisons of empirical base pair coverage for coding DNA sequence regions, and also based on the studies in Gumucio et al. (1992, 1993). There are other, more sophisticated scoring schemes such as PhastCons (Siepel et al. 2005), which may not be as easy to interpret and could be highly variable across different loci which tend to be problematic for plant genome comparisons. In short, we defined our CNSs as stretches of sequences that are contained within a significant orthologous, nonrepetitive segment that are conserved in at least 70% of the species in comparison and at least 6 bp long in length.

### Rice Expression Data

RNA-Seq data on the expression of rice genes were obtained from NCBI Sequence Read Archive, we selected three distinct tissues in different periods, including 20-day leaves, emerging inflorescence, four-leaf stage seedlings (SRA accessions: SRP008821, SRP008821, SRP001787). For each gene by mapping reads to same rice reference genome using the spliced read aligner TopHat v2.1.1 (Trapnell et al. 2009), we calculated the expression in Fragments Per Kilobase of transcript per Million mapped reads (FPKM) using Cufflinks v2.2.1(Trapnell et al. 2010).

### GO Term Enrichment

All enrichment and GO terms reported in this paper were calculated using agriGO (Du et al. 2010). The GO annotation file was retrieved through the Rice Genome Annotation Project (Kawahara et al. 2013). Enrichment was determined using the Fisher’s exact test with correction for multiple testing. Results were considered significant at False Discover Rate (*q* value) <0.01. For simplicity, we only focused on the ontology of Biological Processes (BP). 

## Results

### Computational Pipeline for Whole Genome Alignments

**Fig. 1.**
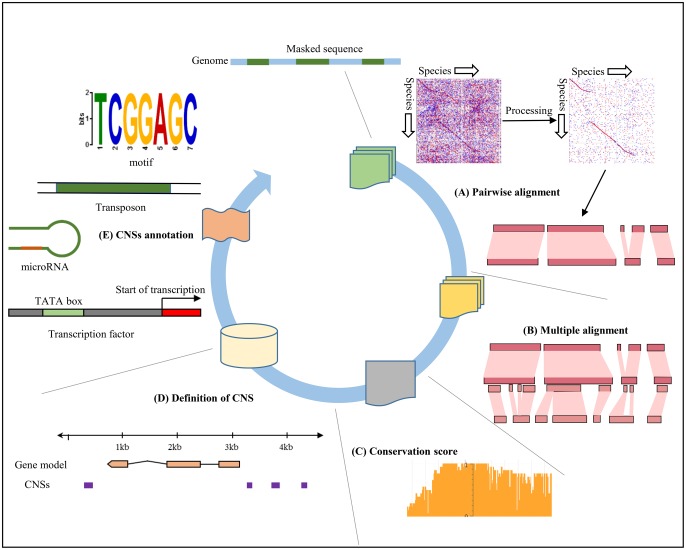
—Our CNSpipeline to identify conserved sequences through genome comparisons. The steps include: (*A*) pairwise genome comparisons followed by masking and chaining; (*B*) merge pairwise alignments into multiple alignments; (*C*) calculation of conservation score of each base; (*D*) prediction of CNSs based on our criteria; (*E*) annotations of CNSs against various genomic elements including known motifs, microRNA, and lncRNA when possible.

An overview of the pipeline is in figure 1. We compiled a set of 17 published grass (Poaceae) genomes, 5 nongrass monocot genomes and *Amborella trichopoda*, for a total of 23 genomes available for comparisons (supplementary table S1, Supplementary Material online). A set of 23-way genome alignments were generated using rice (*O. sativa ssp. japonica*) as the “reference” genome against which all other genomes (“nonreference” genomes) were aligned. Due to the recurring nature of the ancient polyploidization in plants, there are many regions from nonreference genomes that map to the same reference region. In order to enrich for the sequence matches that are likely to be orthologous, that is, which are descended from the same sequence in last common ancestor of the genomes, we only allow a single best region from a nonreference genome to align to a single region of the reference genome. The limitation of a single region per matching species is necessary to remove much noise from the single gene duplications and transposon activities, but largely ignores the paralogous regions derived after the cereal radiation. Some species, like maize or wheat, are known to have had subsequent polyploidy events follow their divergence with reference genome. Since we mostly searched for CNSs from the perspective of the reference genome (rice), it does not matter since either region could indicate conservation in a species affected by recent polyploidy.

Additionally, we only retained local pairwise alignment blocks that belonged to the longest sets of collinear blocks, as defined by “chains” and “nets” (Kent et al. 2003). In short, each genome was aligned to the reference genome using tuned parameters, followed by chaining, netting, and postprocessing into pairwise alignments.

### Phylogenetic Tree Construction Based on Whole Genome Alignments

Phylogenetic trees were constructed covering all the included species in this study to merge pairwise alignments into multiple alignments (Blanchette et al. 2004). Since a common reference is used, the alignments can be stitched together to form multiple sequence alignment blocks in a straightforward manner. For the Poaceae clade, there are a total of 3,759,732 sequence alignment blocks with an average size is 90 bp, with an average of eight species per alignment block.

Building on the multiple alignments, a phylogenetic tree derived from shared sequences can be built (supplementary fig. S1, Supplementary Material online). The overall topology of the tree, constructed from the shared sequences in genome-wide comparisons, confirmed the expected taxa relationships with a few minor differences. The clades of monocots, Poales order, Poaceae family, and Bambusoideae–Ehrhartoideae–Pooideae (BEP) subclades are well separated. However, differences were observed, such as the relationship among the Triticinae species (*Aegilops tauschii, Triticum urartu*, and *Triticum aestivum*) and between *Ananas comosus* and Poaceae. The difference in the Triticinae species may be caused by several polyploidy events in their evolutionary history. In our species tree constructed from our whole genome alignments, *Ananas comosus* is closer to other monocots than to Poaceae, although it is considered in the same Poales order as the grasses (supplementary fig. S1, Supplementary Material online). Additionally, shared sequences were divided into coding sequences and noncoding sequences according to the annotation of reference genome and the noncoding tree showed a similar trend, indicating the overall consistency between the phylogenetic information in the coding versus noncoding sequences.

### Variation in the Distribution of Conserved Elements

**Fig. 2.**
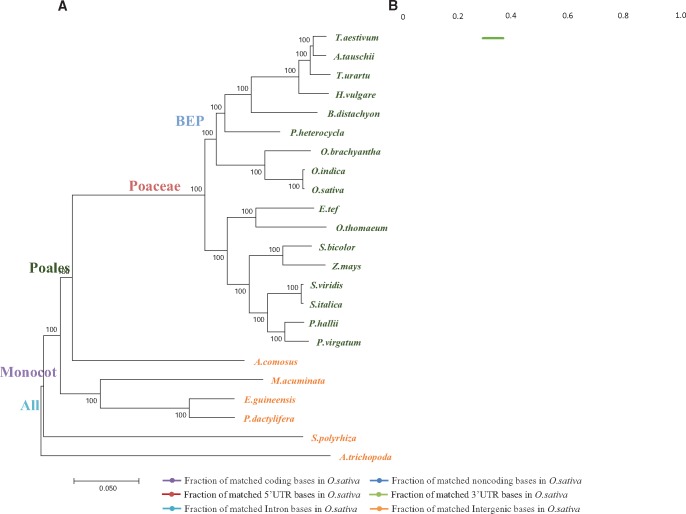
—Phylogenetic tree and base pair coverage based on conserved sequences. (*A*) The phylogenetic tree obtained using shared sequences that are aligned across all genomes. (*B*) Base pair coverage for coding sequence (CDS) and noncoding regions based on the annotated rice gene models.

Results showed that different Poaceae genomes vary greatly in terms of genome size, complexity, assembly quality, and phylogenetic distance from the rice reference (fig. 2). For instance, genome size was observed to vary from 271 Mb in *B. distachyon*, to 6,483 Mb in *T. aestivum* (supplementary table S1, Supplementary Material online)*.* We calculated base pair coverage, or total number of base pairs, for both coding and noncoding sequences (fig. 2*A*). Protein-coding sequences across Poaceae genomes show higher level of conservation, with coverage of conserved sequences staying over 55% of all coding sequences (CDS) in rice (fig. 2*B*). In contrast, the protein-coding sequence coverage for nongrass monocots and *Amborella trichopoda* dropped significantly to <40% due to their large evolutionary distance from rice*.* Additionally, coverage score of noncoding sequences was three times lower than the CDSs, and the coverage score for 5′-UTR was slightly higher than the 3′-UTR (∼40% and 48%, respectively). The lowest coverage score was observed in intergenic regions. It is clear that the coverage of conservation mostly decreased with the increasing phylogenetic distance from the reference genome, as expected.

**Fig. 3.**
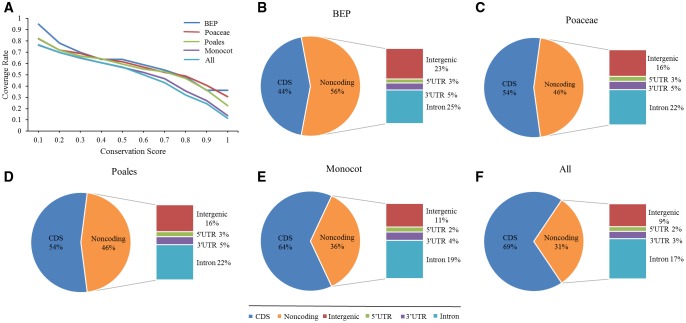
—Estimation of the fraction of sites under selection in the rice genome. (*A*) The fraction of how many species in each clade matched to reference aligned to CDS. Coverage decreased with the increase of the conservation score, but it became stable when the conservation score is close to 0.7. (*B–F*) Breakdown of sites under selection between coding (CDS) and noncoding regions and among different types of noncoding regions in (*B*) BEP, (*C*) Poaceae, (*D*) Poales, (*E*) monocots, and (*F*) all selected genomes.

In order to predict conserved regions in different clades, we calculated a “conservation score” for each base that is equal to the number of species that contain sequences aligned to this site of the reference genome. This conservation score is more straightforward to interpret than alternative scoring methods such as PhastCons (Siepel et al. 2005). To define “conserved” sequences, we chose a score threshold based on the amount of sequence overlapping with the coding sequences in the reference genome, as the coding sequences were largely expected to be conserved. We observed that the overlap decreased with the increase of the conservation score (fig. 3*A*). Based on the base pair coverage for coding DNA sequence regions (fig. 2*B*), any base with a conservation score ≥0.7 is defined as a conserved site, which strikes a balance between sensitivity and specificity of distinguishing coding versus noncoding regions in our experiment.

Finally, we define “conserved sequences” separately for five different clades, BEP, Poaceae, Poales, monocots, and all, in increasing species coverage and divergence level from the rice reference genome. The threshold of 0.7 of conservation score were used across all clades.

### Comparisons between Our CNSpipeline and PhastCons

As a form of validation of our method, we have provided detailed analysis to compare our method with PhastCons. Overall, the number of CDS shared between our method and PhastCons has a reasonably high degree of overlaps (∼60%) in each clade (supplementary fig. S2, Supplementary Material online). Our method shows higher coverage of coding sequence in all clades relative to the PhastCons. Qualitatively, our scoring scheme shows higher sensitivity in both coding and noncoding regions (supplementary table S2, Supplementary Material online). In our visual proofing, PhastCons is inadequate in some regions, such as showing no conservation in some genic regions, or showing uneven conservation scores within known exons, which are inconsistent with the underlying multiple sequence alignments. For example, the exon in gene *LOC_0s01g19340* has extremely low conservation score (supplementary fig. S3, Supplementary Material online) while and the conservation scores for the exons in gene *LOC_0s01g57870* are quite uneven (supplementary fig. S4, Supplementary Material online), in both cases contradicting the underlying sequence alignments.

We chose not to include the comparison to GERP in this study since GERP did not appear to scale with the number of taxa and sites. In addition, results from GERP scores from a 5,000 test set showed an unacceptably low number of conserved domains that could be predicted when compared side-by-side with our conservation score, suggesting that GERP was significantly underpowered when a large number of species are included, at least when tested empirically in the set of genomes that we selected.

### Identification and Distribution of CNSs

Analysis of sites under constraints based on distinct clades showed that, at least 12.05% of the rice genome sequence (45 Mb) have been evolving under constraint in the Poaceae clade, and approximately half of these sequences (20.64 Mb) are located outside the protein-coding regions and hence considered noncoding (fig. 3*C* and supplementary table S3, Supplementary Material online). We divided the constrained noncoding sites into two categories, intergenic sites and gene space that is further consisted of 5′-UTR, 3′-UTR regions, and introns. The proportion of sites under selection was particularly high in introns (22%) and intergenic (16%) (fig. 3*C* and supplementary table S3, Supplementary Material online). We also observed similar trends in other clades (fig. 3). Finally, we recovered a total of 21.12 Mb of CNSs in Poaceae after merging close fragments that are within 3 bp from one another, and removing the fragments with length <6 bp (table 1). The CNSs have an average size ranging from 31 to 55 bp, which is longer than previous studies (Freeling and Subramaniam 2009) (table 1).

**Table 1:** Summary of CNS Distribution in Different Clades

CNS Attributes	BEP	Poaceae	Poales	Monocot	All
Total number	600,632	527,895	533,982	377,266	300,395
Mean length	55 bp	40 bp	40 bp	33 bp	31 bp
Median length	33 bp	24 bp	24 bp	20 bp	18 bp
Total length	33,026,856	21,117,687	21,608,465	12,633,501	9,336,098
Percentage (%) of CNS in intergenic	40.76	35.25	35.83	31.21	30.31
Percentage (%) of CNS in 5′-UTR	5.10	6.56	6.51	6.44	6.07
Percentage (%) of CNS in 3′-UTR	9.17	10.92	10.73	11.26	10.23
Percentage (%) of CNS in intron	44.96	47.28	46.93	51.09	53.39

### Identification of Conserved Genes and Functional Enrichment Analysis of Highly Conserved Genes

**Fig. 4.**
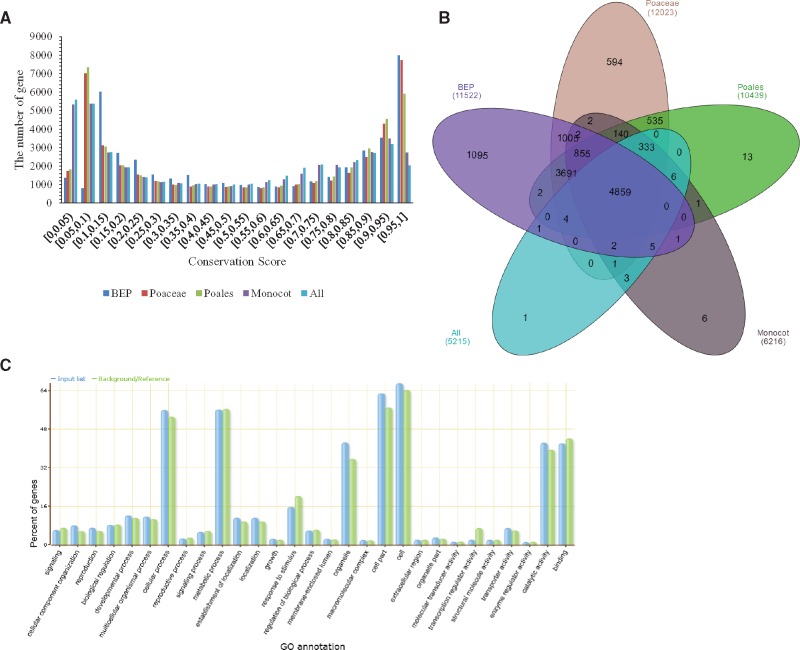
—Highly conserved genes among different clades. (*A*) The distribution of gene conservation scores in different clades. (*B*) Regions are labeled with their respective clade and number of genes that are considered highly conserved. (*C*) Functional categories of highly conserved genes.

To determine whether conserved genes in various different clades showed enrichment of particular functions, we calculated the conserved score for each gene based on the average conservation score of every base within the gene. The distribution of gene conservation scores showed similar trends among each clade (fig. 4*A*). The distribution is very heavy both close to the “no-conservation” (score = 0) and close to “full-conservation” (score = 1), that is, the score almost qualitatively separates the two extremal classes of genes. Further analyses suggested that when we selected the score above, say 0.8, for example, the Gene Ontology (GO) results showed similar enriched classes—so our results do not appear to be too sensitive to the exact cutoffs that we chose, unless we chose an unreasonably low or high cutoff, as determined by the underlying score distribution.

We inferred a set of highly conserved genes (with average conservative score of at least 0.9, that is, showing conservation in 90% of the species that were compared). We also obtained the Gene Ontology (GO) terms of the highly conserved genes among clades. The number of highly conserved genes in different clades vary between 5,215 and 12,023 (fig. 4*B*). Those common genes involved in basic cellular functions, catalytic activity, and binding (fig. 4*C*). Except for common genes, there are many specific conserved genes in each clade contrary to other clades, especially for BEP and Poaceae (1,095 and 594 conserved genes, respectively) (fig. 4*B*). Many of the genes extremely conserved within Poaceae are not so conserved through the monocot clade with the distance of evolution. For example, *waxy* gene, grain number, and cell wall invertase gene related to the production yield and quality appear to be specific to the Poaceae.

### Conservation Score in Gene Structure and Intergenic Regions

Sequences under selective constraints are expected to diverge much slower than the nonfunctional sequences over evolutionary time, therefore, we expect the conservation score of the functional sites to be higher than the nonfunctional sites. In order to test this hypothesis, we calculated the score distribution in different types within the gene, upstream 1 kb of the transcription start site (TSS), downstream 1 kb of the transcription end site (TES) (Alexandrov et al. 2015), and intergenic regions (supplementary fig. S5, Supplementary Material online). The results showed that the distribution of the conservation score tend to show similar trends through different clades. Outside protein-coding transcripts, the score decreased with the increasing physical distance from the TSS (supplementary fig. S5*A*, Supplementary Material online). However, the score was particularly high within protein-coding transcripts and the UTR regions which are located near coding sequences in both BEP and Poaceae. However, the sharply increased score within 350 bp of the TSS suggested that most of the regulatory elements are effectively located within those regions in the rice genome.

Intronic and intergenic bases showed similar trends in distinct clades, and bases located near the TSS or TES seem to be under stronger selective pressure than other intronic or intergenic bases (supplementary fig. S5*B* and *C*, Supplementary Material online). Additionally, compared with other exons, the average score of the first exon and the last exon are low, and the average score of UTR is lower than other exons (supplementary fig. S5*D*, Supplementary Material online). The unevenness of the conservation score reflects the functional landscape of the regions surrounding the coding genes.

### Evidence of Purifying Selection on Conserved Sequences at the Population Level

The conserved sequences were identified through cross-species comparisons, and were determined to be under strong purifying selection over evolutionary time scale. We further asked the question whether these sequences continue to be under strong selection within the rice population. We compared our conserved sequences against the SNP-Seek database of single-nucleotide polymorphisms (SNPs) derived from 3,000 rice genomes (Alexandrov et al. 2015).

**Fig. 5.**
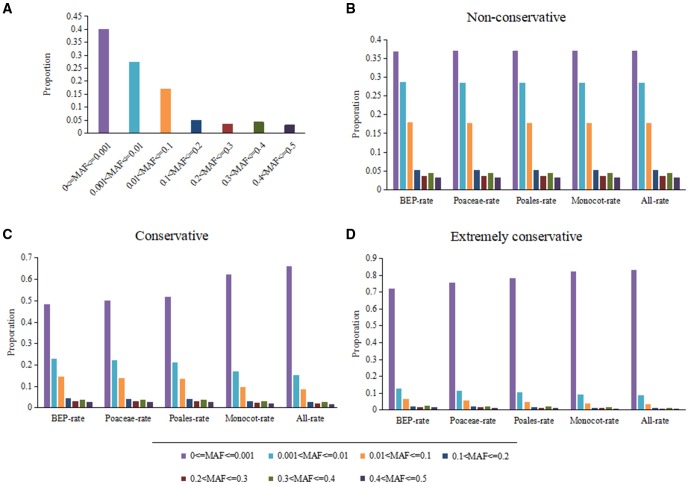
—Evidence of selection on conserved sites in the rice population. Minor allele frequency (MAF) distribution is shown at single polymorphic sites (SNPs) present in 3,000 rice genomes. When summarized at different clade level, they show similar levels of variation in different conserved categories, where the most conserved sites appear to be under the strongest selective pressure.

Constrained sequences show a depletion in SNPs frequency, which is 1.36-fold lower rate than the genome average. Evidence for purifying selection acting on conserved sequences was also found in the minor allele frequency (MAF). MAF indicates frequency of these SNPs occurred within the rice population. Extremely low MAF indicate rare SNPs, which are considered to be less tolerant to mutations than sites with higher MAF. These SNPs were binned according to varying MAF categories (fig. 5*A*). Additionally, we extracted three types of sites based on the conservation: sites that are nonconservative (conservation score from 0.0 to 0.2) (fig. 5*B*); sites that are conservative (conservation score from 0.7 to 0.9) (fig. 5*C*); sites that are extremely conservative (conservation score from 0.9 to 1.0) (fig. 5*D*). This forms the basis of the study of the degree of overlaps between cross-species and intraspecies variation.

Previous studies on maize (Yang et al. 2017) and poplar (Zhang, Zhou, et al. 2016) indicated the negative correlation between the deleteriousness and minor alleles frequency, and results showed low alleles frequency suggesting purifying selection, which were enriched within regions showing evidence of selection and regions of low recombination. Much of the selective constraints are expected to persist regardless of the evolutionary scale, as our results showed. The population frequency of the rare SNPs is clearly correlated with the level of conservation over evolutionary time scale, thus further supporting the functional roles of these sequences, both at the level of different species as well as within the rice diversification.

**Fig. 6.**
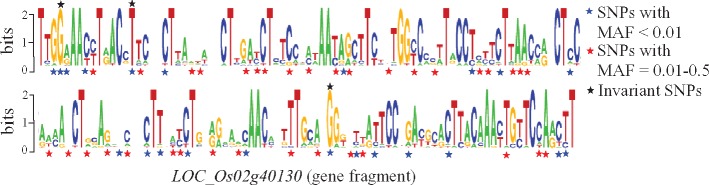
—Sequence logo of one fragment of an exemplar gene harboring SNPs positions that illustrate sites with high intraspecific variation yet low interspecific variation. Blue stars represent the rare SNPs of MAF <0.01. Red stars represent SNPs of MAF >0.01 but <0.5. Black stars represent cross-species “invariant” SNPs that are consistent throughout the multiple sequence alignment blocks across species. This fragment is located in Chr2: 24,293,421–24,293,580 of the rice genome.

The most extreme cases of purifying selection are the “invariant sites,” which could be identified as sites that shared identical bases in the cross-species multiple sequence alignment blocks but referred as SNPs in previous studies. For example, SNPs located in gene *LOC_Os02g40130* show the cross-species “invariant” SNPs that surprisingly show variations in the population although mostly in low frequencies with MAF <1% (fig. 6). We divided the invariant sites into four categories according to their level of impacts on gene function—high, low, moderate, and modifier, based on SnpEff (Cingolani et al. 2012). Results showed that 2.14% (1,479/23,811) of total invariant sites have large impacts (moderate to modifier) on gene function (table 2). We also calculated the percentage effects of those invariant sites according to their types and regions where they are present. We found that exons, downstream, and upstream regions have high concentration of rare SNPs, mostly matching the distribution of the conserved elements that we predicted (table 2).

**Table 2:** Number of Effects by Type in the Rice Genomes Based on the Invariant Sites

Types	Count	Percentage (%)
3′-UTR variant	915	1.29
5′-UTR premature start codon gain variant	91	0.13
5′-UTR variant	643	0.91
Downstream gene variant	19,370	27.37
Intergenic region	1,054	1.49
Intron variant	1,262	1.78
Missense variant	26,790	37.86
Splice acceptor variant	228	0.32
Splice donor variant	313	0.44
Splice region variant	1,124	1.59
Start lost	13	0.02
Stop gained	916	1.29
Stop lost	9	0.01
Stop retained variant	1	0.00
Synonymous variant	4,201	5.94
Upstream gene variant	13,835	19.55

Note.—The invariant sites are the columns with no mismatches based on the multiple sequence alignments across the selected genomes that we reconstructed in this study.

### The Relationship between Intraspecific and Interspecific Variation

We counted the number of substitutions of each gene on the intraspecific (based on population genetics data) as well as on the interspecific level (based on cross-species comparisons). The results show a strong positive correlation (Pearson’s *R *=* *0.43, *P* value = 0) between intraspecific and interspecific variation in all studied clades (supplementary fig. S6, Supplementary Material online).

Despite the overall concordance between the level of intra- and interspecific variation, we found that some sites show abnormally high level of intraspecific variation that appear to have only occurred in rice. We found a total of 645 SNPs sites with extremely high conservation score (identical base in every species compared) but high MAF (high level of mutation frequency) in rice. They are the sites that have “relaxed selection” in rice. For example, one SNP in *OsValRS2* caused virescent to albino phenotypes in seedlings and white panicles at heading but show very strong conservation in other genomes at this site (Wang et al. 2016).

Other genes that show high intra- and low interspecific variations have most of their putative functions related to the disease resistance and receptor-like protein kinase. Genes that show high intraspecific variations may be positively selected in the rice lineage since their functions may be related to the ecological adaptations for rice. Although these genes have high level of intraspecific variation, they are conserved in cross-species comparisons, suggesting the selection on those genes have only recently been relaxed. As an example, we illustrate a portion of a rice gene *LOC_Os02g40130* (fig. 6). In *LOC_Os02g40130*, a total of 555 of SNPs are observed, however, 93 of them show no variations in interspecific multiple alignment. These results suggest that those intraspecific SNPs in rice were resulted from relatively recent positive selection, while being still subject to strong negative selection in other genomes.

Conversely, there are 106 genes conserved only in the rice lineage but are not conserved in other genomes, with their main functions mostly involved in transposon proteins, SCP-like extracellular proteins, and pollen allergen. These genes might have a relatively recent origin but somehow obtained new functions and hence under placed under purifying selection. In-depth studies of those “outlier” genes that have abnormal level of intraspecific variations may ultimately reveal interesting rice biology as well as functional innovations that have occurred in the rice lineage.

### Functional Annotation of CNSs

Many CNSs remained conserved over millions of years of evolution, suggesting that they play vital roles in regulating biological processes such as growth and development. To understand and study the functions of these CNSs, we studied 5,761 rice genes that are located in the 1-kb downstream of the identified CNSs. GO enrichment analysis reveals that these genes are likely to mediate the biological functions, mostly related to a variety of regulatory mechanisms (supplementary table S4, Supplementary Material online), consistent with previous studies reports in vertebrates (Bejerano et al. 2004) and plants (Inada et al. 2003; Thomas et al. 2007). A total of 634 transcription factors are identified in these rice genes, which are highly enriched in the CNS-containing genes. These CNSs potentially act as *cis*-regulators of the genes, often containing motifs that are transcription factor binding sites (TFBS) or enhancers, that is, providing mechanisms for *pre*transcriptional regulation. In addition, we identified 2,785 bigfoot genes (genes with >4 kb of gene spaces and at least have six CNSs) with most of them containing nucleic acid binding function (GO: 0005488).

There are 20% of CNSs identified in Poaceae family that are overlapping with repetitive sequences, including at least 51% of those CNSs contain LTRs (40.67%) and DNA transposon elements (11.17%). This is a bit unusual as transposon sequences are generally expected to have a faster substitution rate. These transposons or transposon fragments may be on the path to become “domesticated” (Sinzelle et al. 2009).

We identified 116 known miRNAs that are part of or overlap our identified CNSs, indicating that some CNSs were also directly involved in *post*transcriptional regulation. For example, we identified that a miRNA (*MIMAT0022865*), which overlapped with a 55-bp CNS that is conserved across all targeted genomes, and had high level of sequence similarity with MIR396E in *B. distachyon* (supplementary fig. S7, Supplementary Material online), which was involved in reprogramming leaf growth during drought stress (Mecchia et al. 2013).

In addition to being located in the vicinity of protein-coding genes, we found that some CNSs have overlapped with published lncRNAs (supplementary table S5, Supplementary Material online), and three of them were specifically expressed during reproduction (Zhang 2014). For example, lncRNA *XLOC_024266* is conserved across 22 monocot genomes, with the exception of *Spirodela polyrrhiza* (supplementary fig. S8, Supplementary Material online). Taken together, a significant portion of the identified CNSs can be annotated as components of *pre*- and *post*transcriptional machineries in the plant cells.

### *Cis*-Regulating CNSs Are Enriched for Specific Sequence Motifs

The study of CNSs enabled the direct identification of various regulatory motifs for different transcription factors by highlighting the genomic regions with putative regulatory function. We have observed a large number of known motifs that are conserved in BEP (384), Poaceae (376), Poales (376), and monocot (369), respectively (supplementary table S6, Supplementary Material online). We found that motifs were significantly (chi-squared test, *P* < 0.01) enriched in the CNS regions versus their occurrences across the entire genome.

**Fig. 7.**
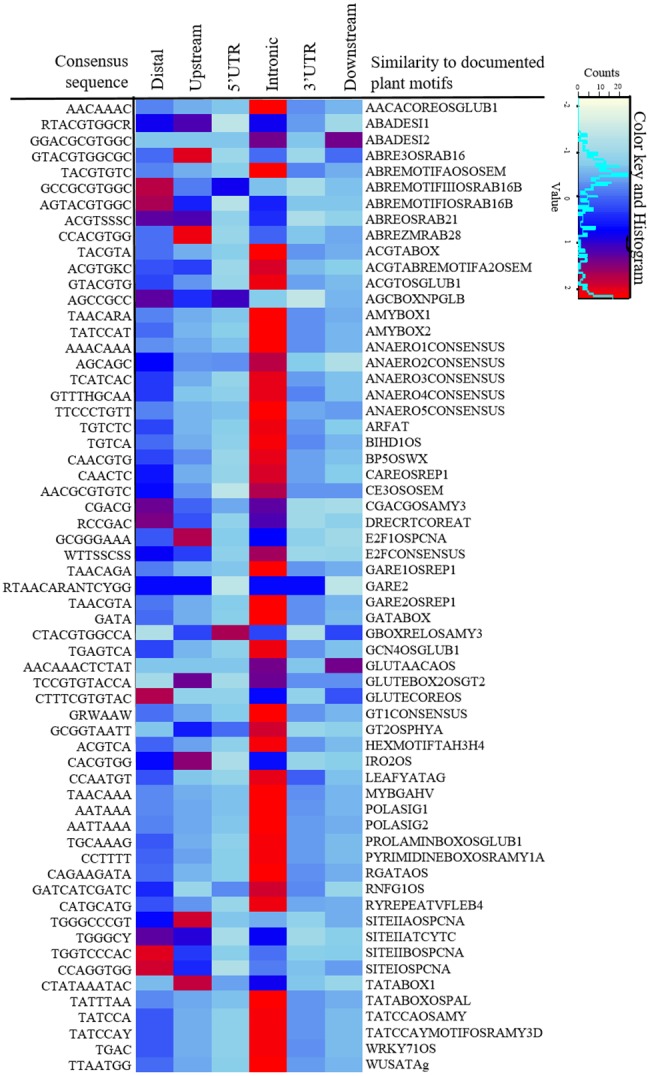
—List of sequence motifs that are enriched in the CNSs. Enrichment is defined as *z*-score >2. Colors in the heat map correspond to different level of fold enrichment. Sequence motif annotations were based on the PLACE database (Higo et al. 1998).

We computed the enrichment of motifs in different types of CNS based on *z*-score. We found that each type of CNS is enriched with at least some motifs registered in the PLACE database (Higo et al. 1998) (fig. 7). Most motifs were associated with the binding preferences of ubiquitous transcription factors. For example, the ABRE3 motif contains a G-box and a novel *cis*-acting element, the beta-phaseolin promoter activity is regulated by the TATA-box motifs (Grace et al. 2004), site-II elements (SITEIIATCYTC) found in the promoter regions of cytochrome which regulate the phosphorylation (*OxPhos*) machinery (Welchen and Gonzalez 2005, 2006) and site-IIa (SITEIIAOSPCNA) elements which are involved in meristematic tissue-specific promotor region of auxin-regulated genes (Kosugi and Ohashi 1997). All of these motifs are highly enriched in our CNS set. Functional validation is still necessary to follow up on many of the enriched motifs in the CNS regions, which have at least indicated putative functions on the basis of sequence conservations. Since we have identified the CNS set through cross-species comparisons, such motifs are likely to carry out critical functions across a wide range of taxa, making them the ideal targets for genetic engineering in a broad range of crops.

### Visualization of Conserved Elements

**Fig. 8.**
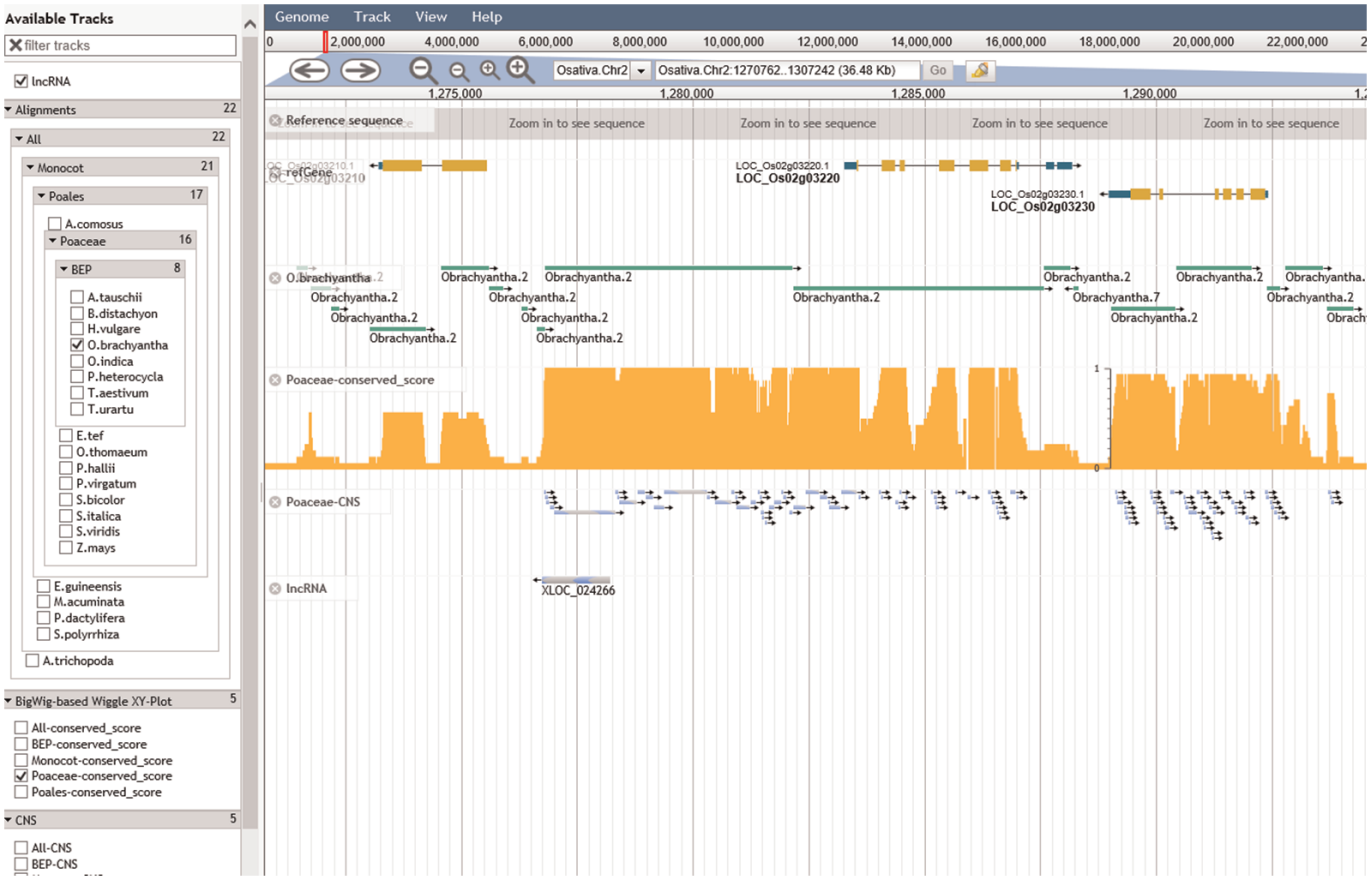
—JBrowse server of the CNS discovery pipeline. Each track represents the alignment between rice (reference) and another query genome. Rectangles represent high-scoring segment pair (HSP) by LAST. The extent of the rectangle indicates the boundaries of the HSP. Chained HSPs outside the coding regions are good candidates of CNSs. As examples, gene *LOC_0s02g03220* and *LOC_0s02g03230* both show high conservation score across the coding region, but they also show high conservation score in the intron (which contains noncoding CNS) and the intergenic regions suggesting the presence of the noncoding CNSs.

In order to visualize the alignments and the associated functional and diversity data directly, we have set up a JBrowse server to host the alignments and conservation tracks obtained in this study (http://angiosperm.org/jsp/ping.html; last accessed January 13, 2018) (fig. 8). The JBrowse instance includes separate tracks including the rice reference genome, rice gene models, a total of 22 pairwise alignments of nonreference genomes against reference genome, SNP variations in the 3,000 rice genomes, and conservation score of each clade that are resulted from this study. The conservation track (wiggle plot) displays conservation score of each base and the conservation score ranges from 0 (no conservation) to 1 (conserved across all genomes compared). For example, gene *LOC_0s02g03220 and LOC_0s02g03230* showed high conservation score across the coding region, but they also show high conservation score in the intron and intergenic region of the genes indicating the presence of CNSs (fig. 8). We also highlight a case of lncRNA that is involved in the sexual reproductive process located in the conserved intergenic region (fig. 8).

## Discussion

Conserved DNA elements still retain high levels of similarity following millions of years of evolution, suggesting that they are subject to strong purifying selection. With the increasing availability of sequenced genomes, many tools and methods have been developed to predict and annotate the protein coding sequences, using both *ab initio* predictions, protein homology as well as transcript evidences (Cantarel et al. 2008). The coding space, while important, reveal only a portion of the gene after all. To better understand all functional features of a gene, we need to better characterize and identify the *cis*-regulatory sequences that mediate the expression of the protein. Available methods are lacking for the identification of CNSs, especially in plants where divergence rate of noncoding sequences is much higher than in the animal genomes. Phylogenetic footprinting and genome-wide comparisons have been proven to be one of the most general approaches to identify the conserved regulatory sequences in genomes of closely related species. Due to the subfunctionalization following recurring whole genome duplication (WGD) events and massive transposon activities in the grass genomes, it is not straightforward to apply the mainstream algorithm used in the comparisons of mammalian genomes.

To investigate the functions of CNSs in grasses, we have developed a computational approach, called CNSpipeline, to exhaustively and accurately identify noncoding DNA elements through the comparisons of whole genomes across species. We have compared our CNSpipeline with PhastCons. Our method is simplistic in that it only counts the multiplicity of the concordant sites in the species included in the comparison. PhastCons takes an alternative approach to the detection of conservation—instead of scoring individual bases, they allow information to be aggregated across adjacent sites using a Hidden Markov Model (Hubisz et al. 2011). Overall, our method showed higher sensitivity of genic regions and also in nongenic regions when compared with the PhastCons. In our visual proofing with JBrowse, PhastCons appears inadequate in many regions, such as underestimating level of conservation in some exons and shows very uneven conservation score within known conserved exons, when compared side-by-side with our method.

Genome-wide comparison of flowering plants has facilitated the annotation of sequences based on the patterns of conservation and to find the novel features that may indicate potential regulatory sequences. In our study, we relied on whole genome alignments across 23 angiosperm genomes that focus mostly on the grass family, and estimated conservative scores every base in several major clades in monocots. We successfully extracted a large number of CNSs in BEP (231,263) followed by the Poaceae (184,247), Poales (105,244), monocots (30,441), and angiosperms (24,536) with average conservation score >0.9 across all sites. The conservations scores showed similar trends of distribution among each clade where we would not expect much variation for a qualitative assessment. The main shift appears to have only involved bins of extremal conservation values for deep comparisons such as across monocots or across angiosperm.

We discovered CNSs located in distal, upstream, 5′-UTR, intragenic, 3′-UTR, and downstream. Many CNSs are associated with various known motifs, miRNA and lncRNA and consequently participate actively in the pre- and posttranscriptional machineries. Compared with the methods that are dependent on experiments, for example, chromatin immunoprecipitation (ChIP) and enhancer assay, our in silico method is inexpensive and yet still powerful to predict these potential *cis*-elements from several species at the whole genome level. Among the inferred CNSs, our results have high level of concordance with previously reported binding sites (Gina et al. 2013). About 90% of the CNSs were identified by the CNS discovery pipeline we built, suggesting that this method is effective and accurate. In addition, our functional enrichment analysis reveals that highly conserved genes are involved in a range of house-keeping functions, especially in basic cellular functions, catalytic activity, and binding, reinforcing the view that these basic cellular functions are under the strongest purifying selection.

To investigate the evolutionary history of CNSs in rice, we analyzed the SNPs from 3,000 rice genomes and evolutionary dynamic analysis of allele frequency showed that there is a high level of correlation between conservation score and invariant sites within the population, indicating that these conserved elements consistently play important adaptive roles within the rice lineage. Similarly, Zhang, Zhou, et al. (2016) also showed that deleterious alleles had significant effects on the population dynamics of poplar as mostly challenged by the anthropogenic climatic changes. Another study on maize also indicated the important role of deleterious alleles for incomplete dominance in explaining heterosis (Yang et al. 2017). Our results suggested that, the specific elements that we identified as “outliers”—variant in rice and invariant in other genomes, or conversely, invariant in rice and variant in other genomes—are particularly interesting targets that illustrate unique aspects in the evolutionary history of the rice genome and could have potential application in population studies and plant breeding.

CNSs conserved across Poaceae differ from animals CNSs due to their association with putative target genes and distal CNSs are relatively in low numbers and much shorter in length in Poaceae than in animals (Margulies et al. 2007). These differences in the distribution of CNSs across various regions may reflect the structural differences of introns and exons between plant and animals. Further functional analysis showed that genes with CNSs in their upstream 1 kb were enriched in GO categories of regulation, and represent a majority of the transcription factors in the genome. This result suggests that CNSs are able to rewire existing regulatory networks via active or inactive regulatory genes. In addition, many of these CNSs overlap with a number of noncoding RNAs with known function, such as lncRNA and micro-RNA, that are currently annotated with important functions in plant genomes.

Recent studies of genome wide comparison of rice suggested a strong positive correlation between the presence of CNSs and open chromatin (Zhang et al. 2012). Another work showed that Arabidopsis homeologs enriched with 5′-CNSs showed lower expression than genes with less CNSs (Spangler et al. 2012). Wang et al. (2015) also found that the *OsSPL16-GW7* regulatory module, which is located in the upstream of *GW7* gene, affected grain shape and quality, but not the production. Furthermore, research on the barley genome identified a microRNA binding site of *HvAP2* gene that affected the shape and size of the spike, showing that once mutation occurs in the binding site of microRNA, it will change the binding ability with microRNA172 and ultimately alter the expression of *HvAP2* gene to generate more grains (Houston et al. 2013). We also identified these studied binding sites as present in our CNS set, confirming the exhaustiveness of our CNS set.

Our large exhaustive CNS data set has provided a valuable set of resources to associate the constrained potions of the noncoding genome with biological functions. Specifically, the CNSs annotated in this study could help researchers to isolate and elucidate the regulation of genes for agronomic traits from many cereal crops with genomes ranging from relatively small genome (e.g., rice) to large and complex genomes (e.g., maize and wheat).

## Supplementary Material

Supplementary data are available at *Genome Biology and Evolution* online.

## Supplementary Material

Supplementary Figures and Tables
