# Supplementary material for: Single-Base Resolution Map of Evolutionary Constraints and Annotation of Conserved Elements across Major Grass Genomes
Source: Genome Biol Evol. 2018 Jan 25;10(2):473–88. doi: 10.1093/gbe/evy006 (PMC5798027; doi:10.1093/gbe/evy006)
Supplement: Supplementary Figures and Tables [file evy006_supp.docx]

***Supplementary Information***

**Single-base resolution map of evolutionary constraint and annotation of conserved elements across grass genomes**

Pingping Liang^1, 2^, Hafiz Sohaib Ahmed Saqib^3, 4^, Xingtan Zhang^1^, Liangsheng Zhang^1^, Haibao Tang^1, *^

Supplementary figures (8 total)
Supplementary tables (6 total)

# Supplementary Figures

**
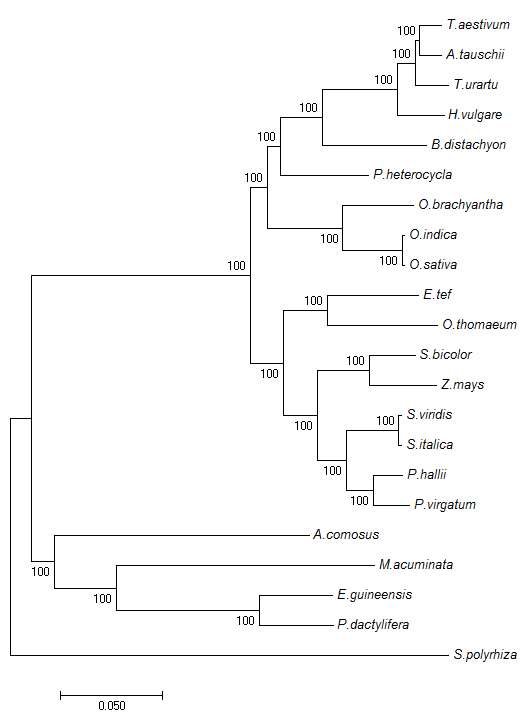
**

**Figure S1. Phylogenetic tree based on shared sequences in the whole genome alignments.**

**
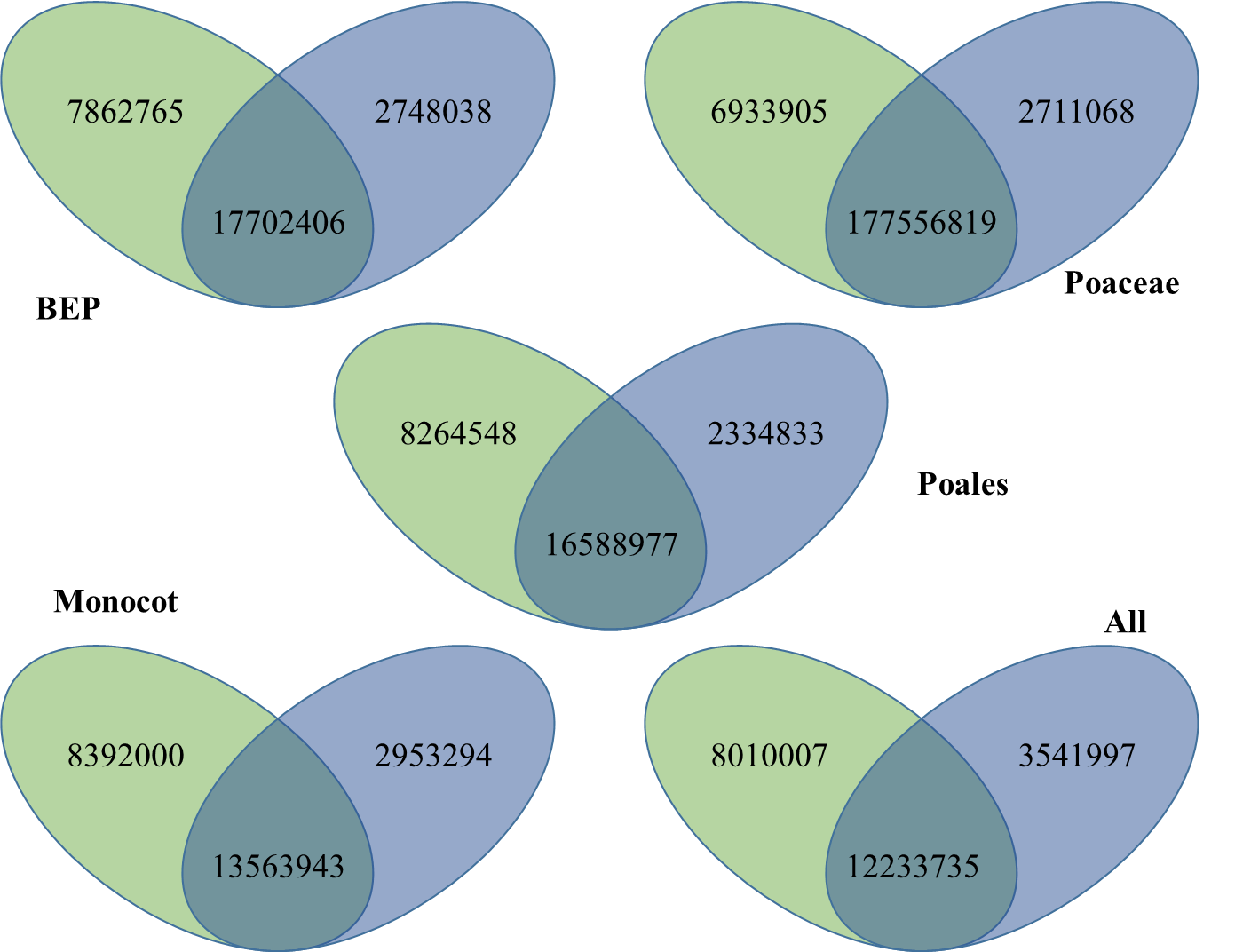
**

**Figure S2. Number of conserved sited predicted from CNSpipeline and PhastCons in different clades.** Green circles represent the number of conservation sites in the CDS of reference genome from our CNSpipeline pipeline. Blue circles represent the number of conservation sites in the CDS of reference genome from PhastCons. Overlapping parts show number of conservation sites in the CDS of reference genome from both method.


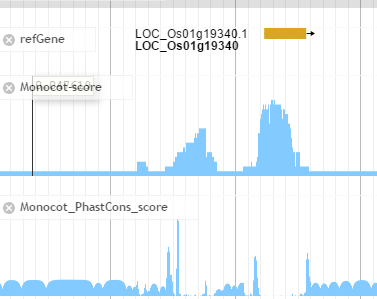


**Figure S3. JBrowse view to compare our scoring scheme vs. PhastCons score in an exemplar gene *LOC_Os01g19340*.** Top track represents the gene model of reference, and the next two tracks represent the conservation score of CNSpipeline and PhastCons, respectively. PhastCons cannot predict the conservation of main exon of the gene *LOC_0s01g19340*.


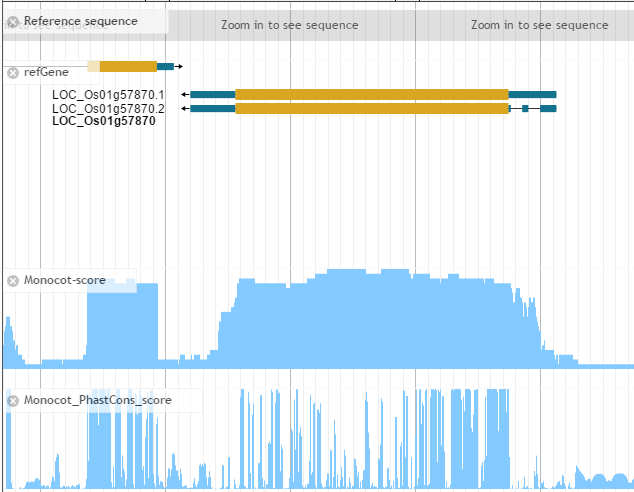


**Figure S4. JBrowse to compare our scoring scheme vs. PhastCons score in an exemplar gene *LOC_Os01g57870*.** Top track represents the gene model of reference, and the next two tracks represent the conservation score of CNSpipeline and PhastCons, respectively. PhastCons shows very uneven conservation scores in CDS of Gene *LOC_0s01g57870*, where multiple sequence alignments showed more even level of conservation.

**
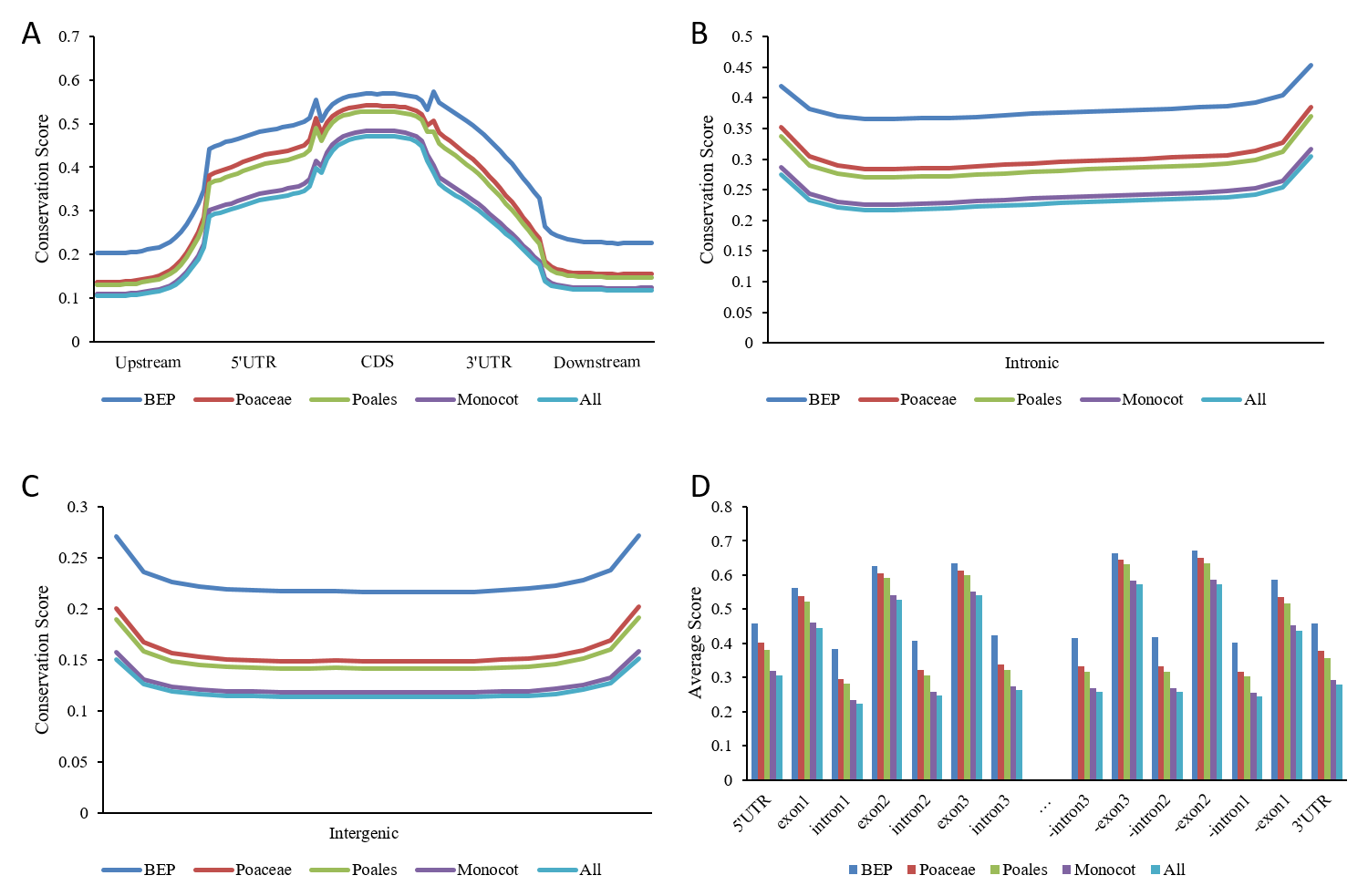
**

**Figure S5. Conservation score distribution within the gene space and intergenic regions according to different conserved scores in different monocot clades. (ABC)** The distribution of scores show similar trends throughout different clades. (**D**) The average score of UTRs, first three exons, last three exons as well as introns of each gene in different clades.


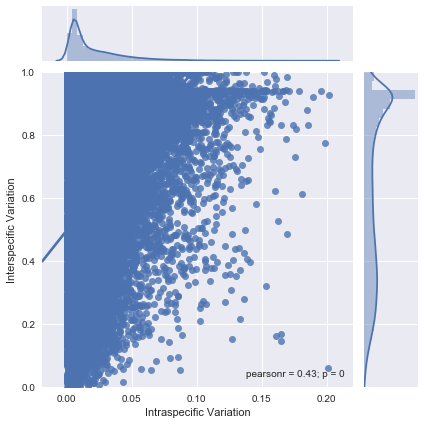


**Figure S6. The relationship between intraspecific variation and interspecific variation.** SNP positions indicate the level of *intra*-specific variation, and the inconsistent bases of multiple alignments indicate the level of *inter*-specific variation. There is a significant positive correlation between the intra-specific and inter-specific variation, as well as ‘outlier’ cases that are interesting targets during the evolutionary history of the rice genome.


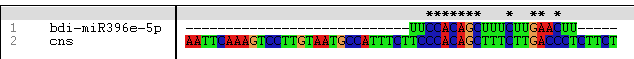


**Figure S7.** **Alignment of a 55bp CNS that includes miRNA (*****MIMAT0022865*) and** **MIR396E in *B. distachyon*.**

**
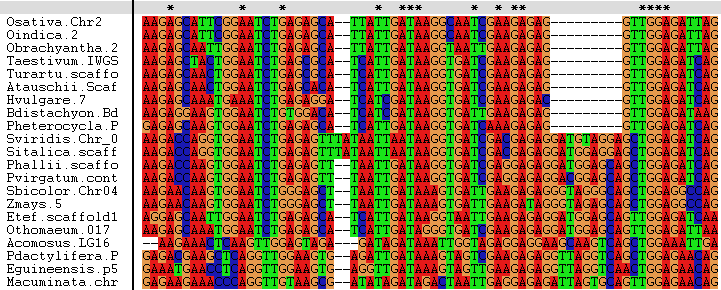
**

**Figure S8. Multiple sequence alignment of one fragment of lncRNA *XLOC_024266* that is conserved across 22 monocot genomes.** The only exception is that the CNS is not found to be conserved in *Spirodela polyrrhiza.*

# Supplementary Tables

**Table S1. Species included in the whole genome alignments in this study.**

| **Name** | **Common  Name** | **Type** | **Nucleotides**  **(bp)** | **N50**  **(bp)** | **CDS aligned (%)^a^** |
| --- | --- | --- | --- | --- | --- |
| *Triticum aestivum* | bread wheat | pseudochr | 6,483,288,884 | 207,910,053 | 64.63 |
| *Triticum Urartu* | red wild einkorn | scaffold | 3,747,163,292 | 85,733 | 55.28 |
| *Aegilops tauschii* | goatgrass | scaffold | 3,313,764,331 | 68,370 | 60.37 |
| *Hordeum vulgare* | barley | pseudochr | 4,045,300,851 | 561,411,686 | 59.75 |
| *Brachypodium distachyon* | purple false brome | pseudochr | 271,163,419 | 59,130,575 | 58.73 |
| *Phyllostachys heterocycle* | moso bamboo | scaffold | 2,051,719,643 | 328,698 | 65.96 |
| *Oryza brachyantha* | wild rice | pseudochr | 260,838,168 | 21,479,432 | 62.85 |
| *Oryza sativa spp. indica* | rice | pseudochr | 427,004,890 | 31,240,961 | 91.05 |
| *Oryza sativa spp. japonica* | rice | pseudochr | 374,471,240 | 29,958,434 | 100 |
| *Eragrostis tef* | tef | scaffold | 672,766,097 | 103,952 | 64.02 |
| *Oropetium thomaeum* | oropetium | scaffold | 243,174,629 | 2,386,382 | 56.89 |
| *Sorghum bicolor* | sorghum | pseudochr | 732,152,042 | 68,658,214 | 60.19 |
| *Zea mays* | maize | pseudochr | 2,067,864,162 | 217,959,525 | 56.42 |
| *Setaria viridis* | green foxtail | pseudochr | 394,903,069 | 46,083,338 | 62.35 |
| *Setaria italic* | foxtail millet | scaffold | 405,737,341 | 47,253,416 | 62.49 |
| *Panicum hallii* | switchgrass (diploid) | scaffold | 556,945,529 | 1,251,710 | 60.78 |
| *Panicum virgatum* | switchgrass (tetraploid) | pseudochr | 1,697,286,449 | 53,011,891 | 65.75 |
| *Ananas comosus* | pineapple | pseudochr | 381,905,120 | 11,759,267 | 37.76 |
| *Musa acuminate* | edible banana | pseudochr | 472,960,417 | 34,148,863 | 36.87 |
| *Elaeis guineensis* | African oil palm | pseudochr | 1,535,150,282 | 1,268,079 | 37.45 |
| *Phoenix dactylifera* | date palm | scaffold | 381,563,256 | 30,480 | 34.38 |
| *Spirodela polyrrhiza* | great duckweed | pseudochr | 145,199,413 | 4,924,802 | 28.4 |
| *Amborella trichopoda* | Amborella | scaffold | 706,332,648 | 4,927,027 | 25.54 |

^a^ “CDS aligned” is the percentage of query sequences aligned to the CDS in the reference.

**Table S2. Comparison between CNSpipeline and PhastCons.**

|  | **The number of conserved bases** | | **Overlapping with CDS** | | **Overlapping with noncoding sequences** | |
| --- | --- | --- | --- | --- | --- | --- |
| Clades | CNSpipeline | PhastCons | CNSpipeline | PhastCons | CNSpipeline | PhastCons |
| BEP | 57,973,906 | 30,549,983 | 25,565,171 | 20,450,444 | 32,408,735 | 10,099,539 |
| Poaceae | 45,131,769 | 27,508,302 | 24,490,724 | 20,267,887 | 20,641,045 | 7,240,415 |
| Poales | 45,985,456 | 25,749,053 | 24,853,525 | 18,923,810 | 21,131,931 | 6,825,243 |
| Monocot | 34,321,308 | 23,386,056 | 21,955,943 | 16,517,237 | 12,365,365 | 6,868,819 |
| All | 29,412,790 | 21,882,772 | 20,243,742 | 15,775,732 | 9,169,048 | 6,107,040 |

**Table S3. Conserved sequence sites in different clades stratified by their locations within the gene space.**

| **Clades** | **Number of bases with conservation score** $\boldsymbol{\geq}$**0.7** | **CDS** | **Non-coding** | **Intergenic** | **5`-UTR** | **3`-UTR** | **Intron** |
| --- | --- | --- | --- | --- | --- | --- | --- |
| BEP | 57,973,906 | 25,565,171 | 32,408,735 | 13,225,677 | 1,656,307 | 2,965,552 | 14,561,199 |
| Poaceae | 45,131,769 | 24,490,724 | 20,641,045 | 7,293,631 | 1,360,908 | 2,241,277 | 9,745,229 |
| Poales | 45,985,456 | 24,853,525 | 21,131,931 | 7,590,531 | 1,381,940 | 2,254,978 | 9,904,482 |
| Monocot | 34,321,308 | 21,955,943 | 12,365,365 | 3,865,688 | 805,766 | 1,374,963 | 6,318,948 |
| All | 29,412,790 | 20,243,742 | 9,169,048 | 2,780,047 | 564,800 | 924,197 | 4,900,004 |

**Table S4. GO enrichment for genes with CNSs in their 1 kb upstream sequences.**

| **GO term** | **Description** | **Number in the conserved gene set** | **Number in the genome** | **FDR  (*q*-value)** |
| --- | --- | --- | --- | --- |
| GO:0010467 | Gene expression | 1136 | 2581 | 3.80E-55 |
| GO:0065007 | Biological regulation | 972 | 2175 | 1.80E-48 |
| GO:0050794 | Regulation of cellular process | 901 | 1983 | 6.70E-47 |
| GO:0050789 | Regulation of biological process | 932 | 2081 | 8.80E-47 |
| GO:0019219 | Regulation of nucleobase, nucleoside, nucleotide and nucleic acid metabolic process | 744 | 1582 | 4.00E-42 |
| GO:0051171 | Regulation of nitrogen compound metabolic process | 744 | 1582 | 4.00E-42 |
| GO:0006350 | Transcription | 783 | 1698 | 4.00E-42 |
| GO:0045449 | Regulation of transcription | 741 | 1576 | 5.80E-42 |
| GO:0080090 | Regulation of primary metabolic process | 769 | 1673 | 5.00E-41 |
| GO:0019222 | Regulation of metabolic process | 782 | 1715 | 7.20E-41 |
| GO:0010556 | Regulation of macromolecule biosynthetic process | 748 | 1616 | 8.00E-41 |
| GO:0009889 | Regulation of biosynthetic process | 748 | 1616 | 8.00E-41 |
| GO:0060255 | Regulation of macromolecule metabolic process | 775 | 1697 | 8.00E-41 |
| GO:0031326 | Regulation of cellular biosynthetic process | 748 | 1616 | 8.00E-41 |
| GO:0031323 | Regulation of cellular metabolic process | 756 | 1639 | 8.00E-41 |
| GO:0010468 | Regulation of gene expression | 752 | 1638 | 4.00E-40 |
| GO:0016070 | RNA metabolic process | 505 | 1180 | 9.40E-21 |
| GO:0051252 | regulation of RNA metabolic process | 377 | 809 | 7.60E-20 |
| GO:0006355 | regulation of transcription, DNA-dependent | 376 | 806 | 7.60E-20 |
| GO:0006351 | transcription, DNA-dependent | 390 | 855 | 2.40E-19 |
| GO:0032774 | RNA biosynthetic process | 390 | 857 | 3.20E-19 |
| GO:0044267 | cellular protein metabolic process | 1024 | 2983 | 5.00E-17 |

**Table S5. List of lncRNAs contained within predicted CNSs.**

| **lncRNA**  **name** | **Strand** | **Chromosome** | **Start** | **End** | **Length** | **Expression pattern** |
| --- | --- | --- | --- | --- | --- | --- |
| XLOC_015509 | - | Chr11 | 18,679,728 | 18,680,040 | 312 | reproductive |
| XLOC_024266 | - | Chr2 | 1,276,742 | 1,278,215 | 1,473 | reproductive |
| XLOC_024928 | - | Chr2 | 9,191,382 | 9,191,582 | 200 | other |
| XLOC_021506 | + | Chr2 | 3,747,160 | 3,747,439 | 279 | reproductive |
| XLOC_034223 | + | Chr4 | 6,670,578 | 6,670,918 | 340 | other |
| XLOC_043729 | - | Chr5 | 16,761,087 | 16,762,092 | 1,005 | other |
| XLOC_045319 | + | Chr6 | 3,370,651 | 3,371,178 | 527 | other |
| XLOC_049821 | - | Chr6 | 26,219,993 | 26,220,356 | 363 | other |
| XLOC_058237 | - | Chr8 | 4,669,832 | 4,670,063 | 231 | other |

**Table S6. The number of motifs enriched with CNSs in each clade based on the PLACE database.**

| **Clades** | **Number of significant motifs in CNSs (all-469)** | **Number of significant motifs in CNSs (rice-75)** |
| --- | --- | --- |
| BEP | 384 | 65 |
| Poaceae | 376 | 62 |
| Poales | 376 | 62 |
| Monocot | 369 | 62 |
| All | 369 | 62 |
